# Testing for *NRAS* Mutations in Serous Borderline Ovarian Tumors and Low-Grade Serous Ovarian Carcinomas

**DOI:** 10.1155/2018/1497879

**Published:** 2018-02-25

**Authors:** Pawel Sadlecki, Dariusz Grzanka, Marek Grabiec

**Affiliations:** ^1^Department of Obstetrics and Gynecology, Collegium Medicum in Bydgoszcz, Nicolaus Copernicus University in Torun, Bydgoszcz, Poland; ^2^Department of Clinical Pathomorphology, Collegium Medicum in Bydgoszcz, Nicolaus Copernicus University in Torun, Bydgoszcz, Poland

## Abstract

The *Idylla NRAS Mutation Test*, performed on the Biocartis Idylla system, is an *in vitro* diagnostic tool for the qualitative assessment of 18 *NRAS* mutations in codons 12, 13, 59, 61, 117, and 146. Low-grade serous ovarian cancer (LGSC) represents less than 10% of all serous ovarian carcinomas. LGSCs are believed to arise from preexisting cystadenomas or serous borderline tumors (SBOTs) that eventually progress to an invasive carcinoma. The molecular analysis of cancer-causing mutations and the development of targeted biological therapies constitute a milestone in the diagnosis and therapy of ovarian malignancies. According to some authors, *NRAS* may be an important oncogene for the progression of SBOT to a frankly invasive disease. The primary aim of this study was to verify if a fully integrated, real-time PCR-based Idylla system can be used for the rapid determination of the *NRAS* mutation status in patients with serous borderline ovarian tumors and low-grade serous ovarian carcinomas. The study included tissue specimens from 12 patients with histopathologically verified ovarian masses, operated on at the Department of Obstetrics and Gynecology, Nicolaus Copernicus University, Collegium Medicum in Bydgoszcz (Poland), between January 2009 and June 2012. The mean age of the study patients was 52.5 years (range 27–80 years). NRAS mutation in codon 13 (G13D, p.Gly13Asp; nucleotide: c.38G>A) was found in one patient, a woman with low-grade serous ovarian carcinoma. To the best of our knowledge, our experiment was the first published study using the novel Idylla NRAS Mutation Test for the evaluation of ovarian tumors in a clinical setting. The Idylla platform is an interesting ancillary first-line rapid and fully automated instrument to detect *NRAS* mutations in SBOTs and LGSCs. However, the clinical usefulness of this method still needs to be verified in larger groups of cancer patients.

## 1. Introduction

Point mutations in cancer cells can be detected with many various methods. The most popular method for the molecular characterization of genetic variants is direct sequencing, which can detect all potential variations, among them, base substitutions, insertions, and deletions. However, direct sequencing has some limitations when applied to clinical samples. First of all, it is not sensitive enough (10–30%) to detect specific point mutations [1]. The analytic sensitivity of this method can be improved by pyrosequencing, high-resolution melting analysis, and real-time or allele-specific PCR assays [2, 3]. Application of direct sequencing as a routine method for cytological diagnosis in a hospital setting requires investment in expensive equipment and implementation of complicated procedures. Another factor limiting the application of this method in everyday clinical practice is long analytical times. This stimulated the search for a simple, rapid, specific, and sensitive method to detect point mutations. Recently, some new molecular assays for the detection of *NRAS*, *KRAS*, *BRAF*, and *EGFR* mutations have become available. These fully automated molecular diagnostic systems are suitable for quantitative allele-specific RT-PCR-based analyses and have been approved by the European Community for *in vitro* diagnostic use (CE-IVD label). As the assays provide high detection rates in *NRAS*, *KRAS*, *BRAF*, and *EGFR* genotyping, they can be used as a standardized method, even at diagnostic centers without a state-of-the-art molecular infrastructure [4, 5]. Further, the testing does not require the involvement of highly skilled personnel, and using this instrument, even pathologists from less experienced laboratories can easily integrate morphological findings with molecular data, being crucial for further diagnostic and therapeutic decisions.

Ovarian cancer is one of the most common and lethal female malignancies worldwide. Regardless of the histological subtype, the standard treatment for ovarian cancer includes staging/debulking surgery and individualized platinum-based adjuvant chemotherapy [6, 7]. Low-grade serous ovarian cancer (LGSC) represents less than 10% of all serous ovarian carcinomas and is less sensitive to conventional platinum-based chemotherapy than high-grade serous ovarian cancers (HGSCs) [8, 9]. LGSCs are believed to arise from preexisting cystadenomas or serous borderline tumors (SBOTs) that eventually progress to an invasive carcinoma. While most SBOTs do not have a typical phenotype of invasive carcinoma, microinvasion is not uncommon. In some studies, up to 60% of LGSCs were associated with SBOTs [10–12]. In line with the dualistic model of ovarian carcinogenesis, type I tumors are formed on a borderline background, from a distinct noninvasive (borderline or proliferating) precursor. Although *KRAS*/*BRAF* and TP53/BRCA mutations are very common in low- and high-molecular grade tumors, respectively, they are not found in all ovarian malignancies; this implies that other, still nonidentified pathway-related events, such as *NRAS* mutations, may be involved. *NRAS* is an established oncogene involved in the pathogenesis of other cancer types, including leukemia and melanoma [13, 14]. According to some authors, *NRAS* may be also an important oncogene for the progression of SBOT to a frankly invasive disease [15]. The molecular analysis of cancer-causing mutations and the development of targeted biological therapies constitute a milestone in the diagnosis and therapy of ovarian malignancies. A few years ago, the classification of ovarian tumors was based mainly on the type of their primary tissue, histopathological, and clinical characteristics. Recently, however, oncological diagnosis has been also expanded to molecular features of cancer cells. Typically, molecular testing is performed whenever the detection of a certain alteration may have an impact on diagnosis and/or prognosis or if a targeted biological therapy is available.

The primary aim of this study was to verify if a fully integrated, real-time PCR-based Idylla system can be used for the rapid determination of *NRAS* mutation status in patients with serous borderline ovarian tumors and low-grade serous ovarian carcinomas.

## 2. Material and Methods

The study included tissue specimens from 12 patients with histopathologically verified ovarian masses, operated on at the Department of Obstetrics and Gynecology, Nicolaus Copernicus University, Collegium Medicum in Bydgoszcz (Poland), between January 2009 and June 2012. The mean age of the study patients was 52.5 years (range 27–80 years). All patients underwent surgical resection adequate for the clinical stage of their malignancy and, whenever necessary, received adjuvant platinum-based chemotherapy in line with current Polish guidelines [16]. The clinical stage of the ovarian tumors was determined in line with the guidelines of the International Federation of Gynecology and Obstetrics Committee on Gynecologic Oncology [17]. Based on histopathological examination of surgical specimens, five lesions were classified as low-grade serous ovarian carcinomas and seven as serous borderline ovarian tumors [18].

Clinicopathological characteristics of the study subjects are summarized in Table 1.

### 2.1. NRAS Mutation Assessment

Molecular studies of formalin-fixed paraffin-embedded (FFPE) specimens were conducted at the Department of Clinical Pathology, Nicolaus Copernicus University, Collegium Medicum in Bydgoszcz. *NRAS* mutation assay is one of the available diagnostic tests (CE-IVD) that can be conducted using the Idylla system (Biocartis, Mechelen, Belgium). The Idylla platform is a fully automated instrument based on a real-time PCR and fluorophore-based detection system, which, depending on the type of the used test cartridge, is suitable for the identification of *NRAS*, *BRAF*, *KRAS*, and *EGFR* mutations. The *Idylla NRAS Mutation Test* is suitable for the qualitative detection of 18 *NRAS* mutations: 5 mutations in codon 12 (exon 2), including p.G12C (c.34G>T), p.G12S (c.34G>A), p.G12D (c.35G>A), p.G12A (c.35G>C), and p.G12V (c.35G>T); 3 mutations in codon 13 (exon 2), among them p.G13D (c.38G>A), p.G13V (c.38G>T), and p.G13R (c.37G>C); 1 mutation in codon 59 (exon 3), A59T (c.175G>A); 4 mutations in codon 61 (exon 3), namely, p.Q61K (c.181C>A), p.Q61L (c.182A>T), p.Q61R (c.182A>G), and p.Q61H (c.183A>C); 1 mutation in codon 117 (exon 4), p.K117N (c.351G>C;c.351G>T); and 2 mutations in codon 146 (exon 4), p.A146T (c.436G>A) and p.A146V (c.437C>T), as well as *NRAS* total and *BRAF* total (wild-type mutations treated as a sample processing control; data not shown by the system).

Each specimen was examined by two independent pathologists to choose the most representative fragment of tumor tissue, with a minimum cellularity for *NRAS*—at least 10%. Then, each 10 *μ*m section was placed between wet filter papers, transferred to an Idylla Test cartridge, and loaded onto the Idylla system. During the test, isolated nucleic acids were separated to 5 PCR chambers via microfluidic channels inside the cartridge (Idylla NRAS; Biocartis). To provide appropriate real-time PCR amplification and detection, all reagents were used in a dry form. Allele-specific targets were identified with a fluorescence-based detection system. A set of parameters describing generated PCR curves, for example, ΔCq value (calculated as the difference between the quantification cycle value (Cq) of the gene control signal and the Cq of the mutant signal), is determined by the Idylla software. The limit of detection (LOD) is defined as the lowest *NRAS* mutation copy number consistently detected in ≥95% of the cases (with 95% confidence), at an allelic frequency of 5% for most prevalent *NRAS* mutations. A sample is classified as mutation-positive if the parameters of the generated PCR curve are within the validated range. Otherwise, the sample is classified as mutation-negative. The results, calculated by the dedicated Idylla software, were available after a 120 min run time [19].

The protocol of the study was approved by the local Bioethics Committee of the Nicolaus Copernicus University, Collegium Medicum in Bydgoszcz (decision number KB 413/2016), and written informed consent was sought from each patient or her next of kin.

Statistical analysis of the results was carried out with the PQStat ver 1.6.4.112 package. Relationships between the presence of *NRAS* mutation and selected clinical parameters were analyzed with the Fisher exact test. Survivals were compared with log-rank, Wilcoxon-Breslow-Gehan, and Tarone-Ware tests. The results were considered significant at *p* < 0.05 and highly significant at *p* < 0.01.

## 3. Results

**Figure 1:**
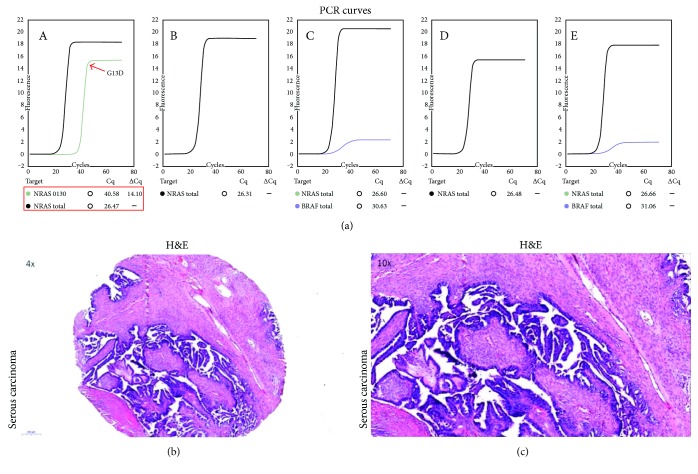
PCR curves from five separated PCR chambers (A–E) for the serous ovarian carcinoma which tested positively for *NRAS* mutation on the Idylla platform (a). Histological specimens from the same tumor, H&E: ×4 (b) and ×10 (c).

**Figure 2:**
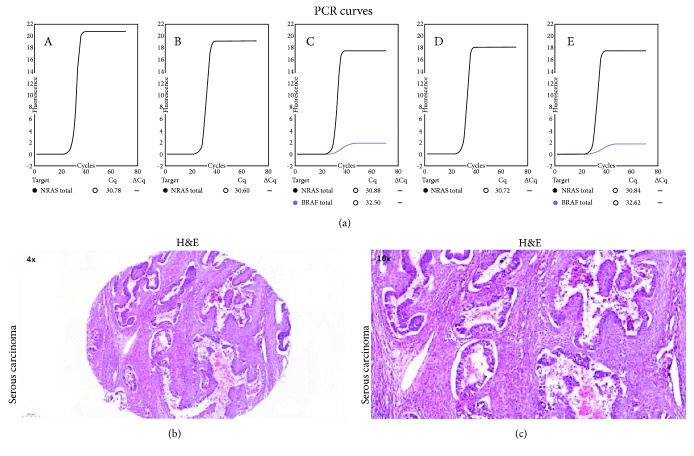
PCR curves from five separated PCR chambers (A–E) for a serous ovarian carcinoma which tested negatively for *NRAS* mutation on the Idylla platform (a). Histological specimens from the same tumor, H&E: ×4 (b) and ×10 (c).

*NRAS* mutation in codon 13 (G13D, p.Gly13Asp; nucleotide: c.38G>A) was found in one patient (8.3%), a woman with low-grade serous ovarian carcinoma. The Cq and ΔCq values were 40.58 and 14.10, respectively. PCR curves with corresponding Cq values for the positive and negative results of the Idylla NRAS Mutation Test are shown in Figures 1 and 2, respectively. Selected clinical characteristics of the woman with *NRAS* mutation and patients who tested negatively for this genetic anomaly are compared in Table 2, and the results of the survival analysis with *NRAS* mutation status as a predictor are shown in Table 3.

## 4. Discussion

The three human *RAS* genes encode four highly related 188- to 189-amino acid proteins, designated as HRAS, NRAS, and KRAS (KRAS4A and KRAS4B). Ras proteins, located on the inner surface of the cell membrane, represent GDP/GTP-regulated switches that convey extracellular signals and are crucial for the intracellular signaling pathways being involved in fundamental cellular processes, such as cell polarity, proliferation, differentiation, adhesion, migration, and apoptosis [20, 21]. Mutations in the neuroblastoma *RAS* viral oncogene homolog (*NRAS)* constitutively activate intracellular signaling through a variety of pathways—most notably, the RAS–RAF–MAPK and PI3K–AKT pathways [22]. Although *NRAS*, *KRAS*, and *HRAS* share some structural and functional similarities, the most frequent *RAS* alterations observed in human malignancies are *KRAS* mutations [23]. The frequency of *RAS* mutants may vary considerably depending on the cancer type, with an estimated incidence of approximately 20% [23]. While amino acid residues G12, G13, and Q61 are the main mutational “hotspots” in colorectal malignancies, also other codons, among them 59, 117, and 146, may be affected [14, 24]. In melanomas, the most commonly mutated isoform of *RAS* is typically found at codons 12 and 61 or, less often, at codon 13. Mutant *NRAS* (Q61) interferes with the GTPase activity of *RAS*, locking it in its active conformation. In turn, *NRAS* (G12) and *NRAS* (G13) mutations affect the Walker A-motif (p-loop) of the protein, decreasing its sensitivity to GTPase-accelerating proteins. Mutations in G12/13 and Q61 can all be classified as activating mutations, yet they affect the NRAS protein in a distinct way, favoring the formation of GTP-bound, active RAS proteins [25, 26]. Testing for *NRAS* mutations is now a standard of care in *KRAS* wild-type colorectal tumors [27]. Nearly 50% of the examined samples will return a wild-type *KRAS* result, and all these specimens should be tested for *NRAS* mutations [28]. Another interesting issue is the occurrence of comutations in RAS–RAF–MAPK pathway-associated genes, which may reflect the molecular heterogeneity of advanced colorectal cancer. Vagaja et al. reported the case of an adenocarcinoma in the caecum, containing a *KRAS*/*NRAS* comutation; the same comutation was also present in the contiguous high-grade tubulovillous adenoma [29]. The early occurrence of comutations implies that *KRAS* and *NRAS* may control different cellular functions; consequently, these two genes may exert a synergistic effect, since *KRAS* is primarily involved in proliferation, whereas *NRAS* is known to regulate cell survival [30].

Our previous study demonstrated that although *KRAS* and *BRAF* mutations never coexisted within the same ovarian tumor, a defect in one of those genes was present in 6 out of 7 SBOTs examined with the Idylla Mutation Test [31]. Ovarian malignancies acquire *KRAS* and *BRAF* mutations at very early stages of their progression, not infrequently before the SBOT stage, and additional driving events, such as the occurrence of *NRAS* mutations, have been postulated to facilitate the progression [15]. Xing et al. analyzed the *NRAS* mutation status of SBOTs, noninvasive LGSCs, and invasive LGSCs; interestingly, they did not find a *NRAS* mutation in either SBOTs or noninvasive LGSCs. According to these authors, the low prevalence of *NRAS* mutations implies that this gene may play a limited role in the development of LGSC [32]. However, in the study conducted by Emmanuel et al., activating *NRAS* mutations were found in 5 out of 58 (9%) invasive serous epithelial ovarian carcinomas coexisting with a serous borderline tumor and in none of the 53 isolated SBOTs. The results of the same study suggested that Ras pathway mutations found in serous ovarian tumors may not be equivalent in terms of their pathogenicity [15]. This hypothesis seems to be also supported by the results of our present study, in which *NRAS* mutation was found in only one LGSC (8.3%) and in none of SBOTs. We also identified *NRAS* mutation as a significant predictor of worse survival, and although our sample was too small to formulate any ultimate conclusions in this matter, still this association needs to be reported. However, the small size of our study group and lack of statistically significant associations between *NRAS* mutation status and histological type of the tumor put the importance of this finding into question.

*NRAS* mutations are also found in melanoma, colorectal cancer, follicular thyroid cancer, and acute myeloid leukemia [33–36]. In melanomas, *NRAS* mutations occur at a fairly consistent rate of 15–20% [37]. In melanoma patients, the presence of *NRAS* mutation was identified as a predictor of poorer outcomes and turned out to be associated with worse median overall survival (OS) [38]. Moreover, *NRAS* may serve as a biological marker to identify melanoma patients who can benefit from targeted therapies [39]. In colorectal cancer, *NRAS* mutations are found less often, in up to 4% of the cases, and also correlate with less favorable clinical outcome, enforcing the use of special treatment strategies [40, 41]. For example, mutations within EGFR-signaling axis molecules, such as *KRAS*, *NRAS*, *BRAF*, and *PIK3CA*, predict resistance to anti-EGFR therapies [42]. Moreover, *NRAS* mutations are postulated to promote the malignant behavior of cancer cells in advanced colorectal malignancies; the results of both *in vitro* and animal studies suggest that this process can be reverted by silencing mutated codons [43]. These data on the effectiveness of targeted therapies in various malignancies harboring *NRAS* mutations justify further research on the role of these genetic anomalies in ovarian cancer [44].

The diagnostic platform Idylla seems to be an interesting alternative for the time-consuming and costly next-generation sequencing (NGS) techniques used for point mutation testing. The Idylla NRAS Mutation Test, performed on the Biocartis Idylla system, is an *in vitro* diagnostic tool for the qualitative assessment of 18 *NRAS* mutations in codons 12, 13, 59, 61, 117, and 146 [19]. In the case of multiple mutations, only the one with the lowest ΔCq value will be reported. Unlike other currently available technologies, the Idylla Mutation Test does not require manual preprocessing of the sample (deparaffinization, digestion of FFPE tissue, or DNA extraction), since all these procedures are integrated within a single-use cartridge [45]. Instead, whole FFPE tissue sections or macrodissected FFPE specimens are inserted into the cartridge and processed by the Idylla system; also, further stages, that is, real-time PCR-based mutation detection and result reporting, are fully integrated and automated [46]. Due to such characteristics of the platform, neither additional molecular infrastructure nor highly skilled personnel are required to perform the test. The accuracy of mutation assessment depends on several factors, such as tissue volume, DNA quality, and percentage of tumor cells, as well as on the specificity and sensitivity of a given test system [47]. According to some authors, the quality of the results obtained with the Idylla Mutation Test may be also influenced by the time elapsed between the sampling and the testing date. Weyn et al. observed a statistically significant association between the age of the samples that have been collected more than 4 years earlier and the likelihood of obtaining an invalid result with the Idylla KRAS Mutation Test [48]. Moreover, it should be remembered that currently available Idylla systems were designed to detect some specific mutations in *NRAS*, *KRAS*, *EGFR*, and *BRAF* genes. Consequently, Idylla Mutation Tests are not capable of detecting each rare genetic defect; the presence of such rare mutations needs to be confirmed with ancillary molecular analyses, to prevent an inaccurate diagnosis and to implement the most effective treatment.

To the best of our knowledge, our experiment was the first published study using the novel Idylla NRAS Mutation Test for the evaluation of ovarian tumors in a clinical setting. The Idylla platform is an interesting ancillary first-line, rapid, and fully automated instrument to detect *NRAS* mutations in SBOTs and LGSCs. However, the clinical usefulness of this method still needs to be verified in larger groups of cancer patients.

## Figures and Tables

**Table 1:** Clinicopathological characteristics of the study subjects.

lp	Histological type	Age	Menopausal status	Bilateral	Size Left/right (cm)	Stage	Mutation NRAS
1	Cystadenoma papillare serosum proliferans	48	Premenopausal	+	13/10	IC	No
2	Cystadenoma papillare superficiale proliferans	27	Premenopausal	−	18	IA	No
3	Cystadenoma papillare serosum proliferans	47	Premenopausal	−	9	IA	No
4	Cystadenoma papillare serosum proliferans	43	Premenopausal	+	7/6	IB	No
5	Cystadenoma papillare serosum proliferans	35	Premenopausal	−	5	IA	No
6	Cystadenoma papillare serosum proliferans	72	Postmenopausal	−	9	IA	No
7	Cystadenoma papillare serosum proliferans	60	Postmenopausal	+	4/5	IB	No
8	Cystadenocarcinoma papillare serosum	59	Postmenopausal	−	8	IA	No
9	Cystadenocarcinoma serosum	50	Premenopausal	+	6/9	IB	Yes
10	Cystadenocarcinoma serosum	66	Postmenopausal	−	23	IA	No
11	Cystadenocarcinoma papillare serosum	80	Postmenopausal	−	12	IA	No
12	Cystadenocarcinoma serosum	43	Premenopausal	−	7	IC	No

**(a):** 

*NRAS* mutation	Death				Fisher test
	No		Yes		
	*N*	%	*N*	%	
Negative	11	100%	0	0%	0.0833
Positive	0	0%	1	100%	

**(b):** 

*NRAS* mutation	Histological type				Fisher test
	Borderline		Serous		
	*N*	%	*N*	%	
Negative	7	100%	4	80%	0.4167
Positive	0	0%	1	20%	

**(c):** 

*NRAS* mutation	FIGO stage				Fisher test
	IA		Other		
	*N*	%	*N*	%	
Negative	7	100%	4	80%	0.4167
Positive	0	0%	1	20%	

No significant relationships were found between *NRAS* mutation status, mortality, histological type, and FIGO stage of the tumor.

**Table 3:** Results of survival analysis with *NRAS* mutation status as a predictor.

Idylla *NRAS*	Log-rank test	Wilcoxon-Breslow-Gehan test	Tarone-Ware test
*χ* ^2^ statistic	7.2735	6.8182	7.0455
Degrees of freedom	1	1	1
*p*-value	0.0070	0.0090	0.0079

Irrespective of the type of statistical test, the presence of *NRAS* mutation was identified as a significant predictor of worse survival (*p* < 0.01).
